# Supplementary material for: Is Interstitial Chemotherapy with Carmustine (BCNU) Wafers Effective against Local Recurrence of Glioblastoma? A Pharmacokinetic Study by Measurement of BCNU in the Tumor Resection Cavity
Source: Brain Sci. 2022 Apr 28;12(5):567. doi: 10.3390/brainsci12050567 (PMC9139119; doi:10.3390/brainsci12050567)
Supplement: Supplementary file 1 [file brainsci-12-00567-s001.zip › brainsci-1708109-supplementary.pdf]

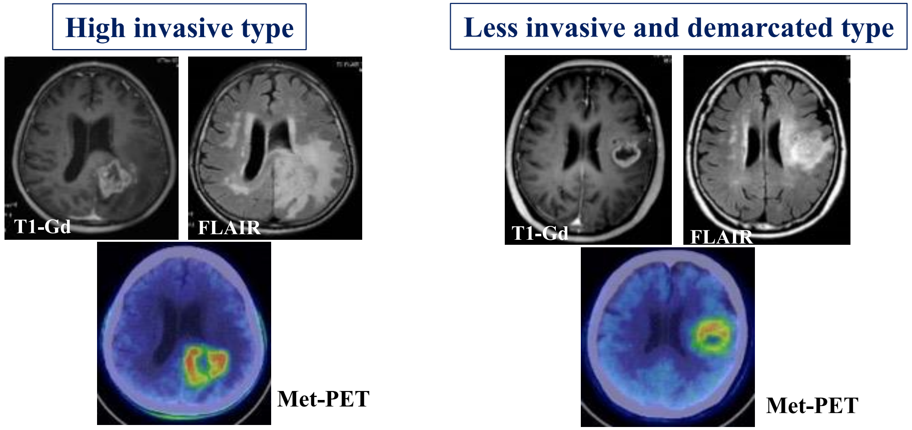

Criteria of two phenotypes of GBM on MRI and Met-PET

Features on MRI/Met-PET	High-invasive	Less-invasive/demarcated
1. Tumor margin	irregular	demarcated
2. Enhancement of margin wall	weak~moderate/ heterogeneous	intense/ homogeneous
3. Peritumoral edema	diffuse	focal
4. Area of Gd+ vs Met-PET (TNR 1.4)	Gd << Met-PET	Gd ≤ Met-PET

Figure S1. Imaging phenotype on magnetic resonance imaging (MRI) in 12 GBMs. Representative images of two phenotypes are shown in the upper panel. Six patients showed the highly invasive type of tumor, and the remaining six patients presented with a less invasive and relatively well-demarcated type of tumor.

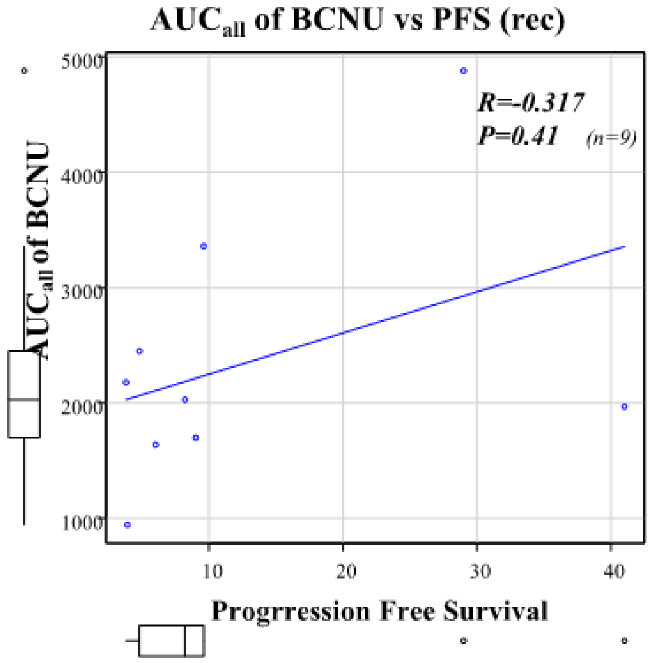

Figure S2. Spearman's regression analysis showing no correlation between PFS of recurrent GBM and AUC_{all} of BCNU in 9 patients.

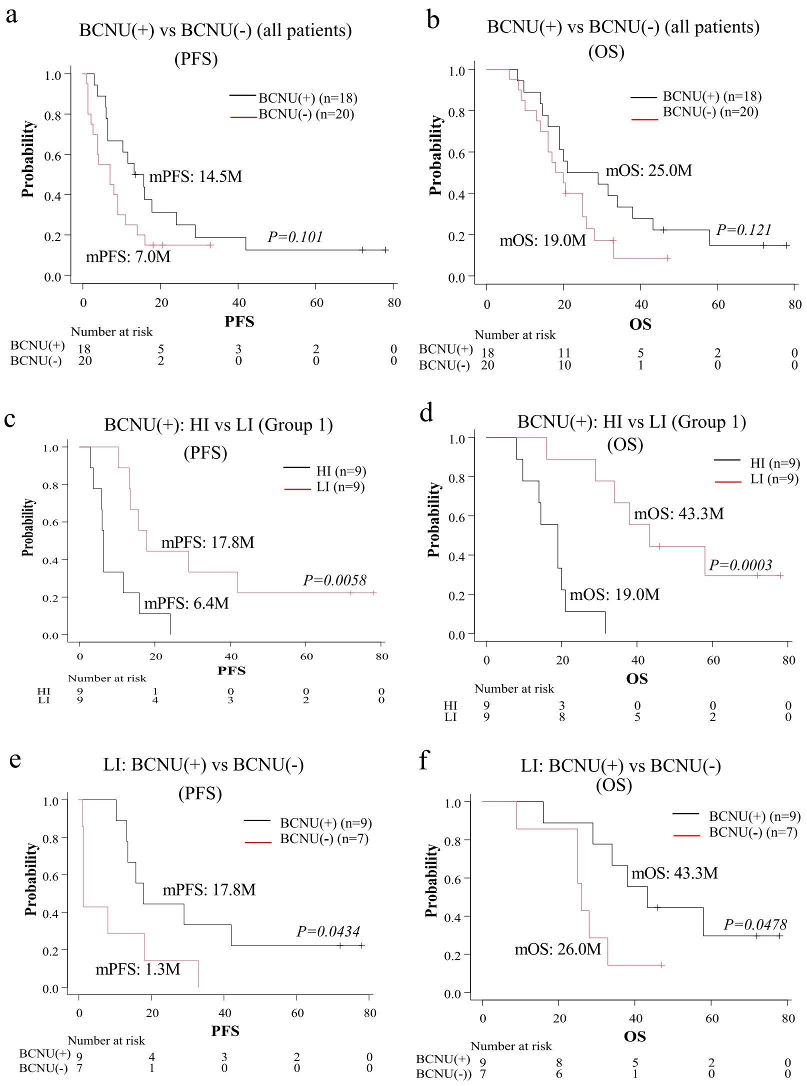

Figure S3. Kaplan-Meier survival curves showing PFS **(a)** and OS **(b)** in 38 GBM patients treated with BCNU wafers (Group 1, *n*=18) and those without BCNU wafers (Group 2, *n*=20). No significant differences in PFS or OS between Group 1 and Group 2 were found. Kaplan-Meier survival curves showing PFS **(c)** and OS **(d)** in the two phenotypes of GBM patients in the group 1. Patients with low-invasive, demarcated type GBM (LI, *n*=9) showed significantly longer PFS and OS than those with highly invasive type GBM (HI, *n*=9) (mPFS; LI: 17.8M vs HI:6.4M, *p*=0.0058, mOS; LI:43.3M vs 19M, *p*=0.0003). Kaplan-Meier survival curves in the patients with a low-invasive type GBM. Treatment with BCNU wafers provided significantly longer PFS **(e)** and OS **(f)** than that without BCNU wafers (mPFS; BCNU+: 17.8M vs BCNU-: 1.3M, *p*=0.0434, mOS; BCNU+: 43.3M vs BCNU-: 26M, *p*=0.0478).

Table S1. Oligonucleotide primers used for qRT-PCR.

Genes	Sense/antisense
CD44	5'-AGAAGGTGTGGGCAGAAGAA-3'
	5'-AAATGCACCATTTCCTGAGA-3'
GAPDH	5'-CAGTCAGCCGCATCTTCTTT-3'
	5'-TGACGGTGCCATGGAATTTG-3'

Table S2. Concetrations of carmustine (BCNU) in the cerebral spinal fluid within the tumor resection cavity for 30 days after operation in 15 patients with malignant gliomas treated with BCNU wafer.

Sampling point (day)	Observed values (ng/mL)														
	**G1**	**G2**	**G3**	**G4**	**G5**	**G6**	**G7**	**G8**	**G9**	**G10**	**G11**	**G12**	**G13**	**G14**	**G15**
0.0417	NS	NS	NS	NS	NS	NS	NS	NS	347	346	755	626	494	298	1660
1	NS	NS	NS	NS	NS	NS	NS	NS	990	123	213	538	180	23.5	242
3	101	193	233	269	93.9	481	170	596	80.6	234	233	90.6	22.2	47.5	734
7	124	78.9	42	NS	671	301	66.4	16	184	139	190	158	62.8	29.9	405
14	0.2	2	0.224	6.05	74.8	1.53	10.8	0.201	23.4	0.625	2.54	15	5.78	0.1	55.2
30	BLQ	BLQ	0.15	0.25	0.704	0.182	0.132	BLQ	NS	NS	NS	NS	NS	NS	NS
AUC (0-30day) (ng· d/mL)	2030	1740	1640	1970	4880	3360	1090	2180	2970	1820	2450	1700	945	491	5810

NS: no sample

BLQ: below the lower limit of quntification
